# Fibrotic NASH Index (FNI) Is Associated with Long‐Term Mortality in Individuals with Type 2 Diabetes and MASLD

**DOI:** 10.1111/liv.70676

**Published:** 2026-05-13

**Authors:** Ilaria Barchetta, Flavia Agata Cimini, Sara Dule, Giulia Passarella, Alessandro Oldani, Maria Neve Hirsch, Marta Cirelli, Marta Alfano, Maria Gisella Cavallo

**Affiliations:** ^1^ Department of Experimental Medicine Sapienza University Rome Italy

**Keywords:** cardiovascular disease, fibrosis, MASH, MASLD, mortality, non‐invasive tests, type 2 diabetes

## Abstract

**Background and Aim:**

Metabolic dysfunction‐associated steatotic liver disease (MASLD) frequently coexists with type 2 diabetes (T2D) and increases cardiovascular disease (CVD) risk, with hepatic fibrosis being the main determinant of mortality. Existing non‐invasive fibrosis scores often include age and may be less informative in early‐stage disease or long‐term follow‐up. This study investigated the association between the Fibrotic NASH Index (FNI), an age‐independent marker and long‐term all‐cause mortality in T2D.

**Methods:**

We conducted a retrospective longitudinal study in a cohort of 174 individuals with T2D (baseline age 67.6 ± 11.4 years; BMI 29.3 ± 5.2 kg/m^2^; HbA1c 7.1% ± 1.7%) enrolled in 2000–2001. Complete mortality data at 20 years were available for all the study participants. Hepatic steatosis was assessed by ultrasound, and fibrosis risk was estimated using FNI; FIB‐4 and APRI were also calculated. Baseline metabolic, biochemical and clinical data were recorded.

**Results:**

At baseline, steatosis was present in 78% and high fibrosis risk in 44% of participants. After 20 years, mortality reached 39.7%. The FNI‐defined fibrosis risk was significantly associated with the vital status at 20 years in both univariate and multivariable models adjusted for age, sex, BMI, lipids, renal function, steatosis, diabetes' duration and CVD history (OR 11.75; 95% CI: 2.11–65.50; *p* = 0.005), whereas neither FIB4 nor APRI retained a significant association after multivariable adjustment (FIB‐4: OR 1.50; 95% CI: 0.52–4.37; *p* = 0.45; APRI: OR 1.49; 95% CI: 0.29–7.53; *p* = 0.63).

**Conclusion:**

FNI‐defined liver fibrosis risk is independently associated with long‐term mortality in individuals with T2D. Incorporating non‐invasive, age‐independent fibrosis assessment may help improve early risk stratification and guide personalised management in dysmetabolic populations.

## Highlights

- MASLD is highly prevalent in patients with type 2 diabetes and contributes to long‐term mortality risk.
- In this 20‐year follow‐up cohort, all‐cause mortality reached nearly 40%.
- Fibrosis risk assessed by the Fibrotic NASH Index (FNI) was independently associated with mortality, outperforming FIB‐4 and APRI.
- The age‐independent fibrosis index FNI may improve early risk stratification in type 2 diabetes.

## Lay Summary

Metabolic fatty liver disease (MASLD) is linked to worse health outcomes in people with type 2 diabetes, especially when liver fibrosis occurs. However, identifying patients at higher risk is challenging because commonly used non‐invasive tests may be less reliable in this population. In this study, we found that the Fibrotic NASH Index (FNI), an accurate score for fibrosis prediction in people with metabolic disease, is associated with long‐term risk of death independently of traditional risk factors. FNI calculation could help the identification of high‐risk individuals earlier and tailor their care more effectively.

**Abbreviations**
ACME: average causal mediation effect
ADE: average direct effect
ADO: oral antidiabetic drugs
ApoB: apolipoprotein B
APRI: AST to Platelet Ratio Index
ASCVD: atherosclerotic cardiovascular disease
CKD‐EPI: Chronic Kidney Disease Epidemiology Collaboration equation
CVD: cardiovascular disease
DBP: diastolic blood pressure
FIB‐4: fibrosis‐4 index
FNI: fibrotic NASH Index
HDL: high‐density lipoprotein
HDL‐c: high‐density lipoprotein cholesterol
HOMA‐IR: Homeostasis Model Assessment of Insulin Resistance
LDL: low‐density lipoprotein
MASH: metabolic dysfunction‐associated steatohepatitis
MASLD: metabolic dysfunction‐associated steatotic liver disease
NAFLD: non‐alcoholic fatty liver disease
NASH: non‐alcoholic steatohepatitis
NFS: NAFLD Fibrosis Score
SBP: systolic blood pressure
T2D: type 2 diabetes

## 1 Introduction

Metabolic dysfunction‐associated steatotic liver disease (MASLD), previously named non‐alcoholic fatty liver disease (NAFLD), has become the most prevalent chronic liver disease worldwide, affecting up to 25%–30% of the global adult population [1]. Its prevalence rises dramatically in individuals with components of the metabolic syndrome, particularly type 2 diabetes mellitus (T2D), where estimates suggest that up to 70%–80% of patients may present with hepatic steatosis [1]. This close relationship reflects the shared pathophysiological mechanisms underlying both conditions, including insulin resistance, chronic low‐grade inflammation and adipose tissue dysfunction [2, 3].

Beyond representing a hepatic manifestation of metabolic syndrome, MASLD has been increasingly recognised as a systemic disease, with repercussions that extend far beyond the liver. Individuals with MASLD, especially those with coexisting T2D, face a significantly increased risk of cardiovascular disease (CVD), chronic kidney disease and extra‐hepatic malignancies [4, 5]. Traditionally, cardiovascular complications have been considered the leading cause of mortality in this population. However, recent longitudinal data suggest that liver‐related events and all‐cause mortality also rise substantially in the presence of progressive liver fibrosis [6].

Among the histological features of MASLD, fibrosis – not steatosis or inflammation – is now established as the key determinant of clinical outcomes [7]. Fibrosis stage correlates directly with the risk of liver decompensation, hepatocellular carcinoma and death. Importantly, hepatic fibrosis also acts as an independent predictor of non‐liver‐related events, including cardiovascular mortality and overall survival, regardless of the degree of metabolic control or presence of diabetes‐related complications [8].

In individuals with T2D, MASLD with advanced fibrosis is associated with a more aggressive metabolic phenotype, impaired glycemic control and increased prevalence of both microvascular and macrovascular complications [3, 9, 10]. Moreover, fibrosis may contribute to insulin resistance and pancreatic β‐cell dysfunction, thereby aggravating diabetic progression in a feed‐forward loop [11, 12]. As such, liver fibrosis in the setting of MASLD should no longer be viewed as a collateral finding but rather as an integral component of systemic metabolic deterioration.

Despite the clinical relevance of fibrosis, its diagnosis in routine care remains challenging due to the invasiveness of liver biopsy and the limited use of non‐invasive tools [13]. Several non‐invasive scores, such as the Fibrosis‐4 index (FIB‐4) [14, 15] and the AST to Platelet Ratio Index (APRI) [16], have been developed to estimate fibrosis risk. However, these scores include age as a variable, which may affect their prognostic accuracy, particularly in elderly patients and during long‐term follow‐up. Additionally, they were primarily validated in populations with advanced liver disease, potentially limiting their sensitivity in individuals without clinically manifest fibrosis [14, 15, 16].

The Fibrotic NASH Index (FNI) is a recently introduced, accurate and cost‐effective non‐invasive marker specifically developed to screen for severe liver disease in individuals with metabolic risk factors from the general population and diabetes specialised care setting [17, 18, 19, 20]. Indeed, the FNI estimates fibrosis risk without incorporating age and has shown good diagnostic performance in dysmetabolic populations with a low prevalence of advanced liver disease, especially individuals with T2D, independent of glucose control and diabetes duration [18]. It has been also shown to outperform other non‐invasive tests, such as the FIB‐4, APRI, NAFLD fibrosis score (NFS) and the Hepatic fibrosis score (HFS), in predicting the resolution of significant fibrosis following bariatric surgery [19]. The FNI is based on three easily available laboratory parameters – aspartate aminotransferase (AST), glycosylated haemoglobin (HbA1c) and high‐density lipoprotein cholesterol (HDL‐c) – each weighted according to its relative contribution to predicting fibrotic MASH. Among these, HbA1c and AST have the strongest influence, while HDL‐c contributes inversely to the score. However, evidence regarding its ability to predict long‐term outcomes remains limited.

Therefore, the aim of this study was to evaluate the long‐term prognostic value of the FNI in individuals with T2D, assessing its association with 20‐year all‐cause mortality independently of established cardiovascular risk factors. By focusing on a simple, age‐independent fibrosis marker, we aimed to explore its utility for early risk stratification in dysmetabolic populations without clinically advanced liver disease.

## 2 Methods

### 2.1 Study Design and Participants

This is a retrospective cohort study with a 20‐year follow‐up assessing all‐cause mortality. It involved 174 men and women with established T2D who attended the outpatient metabolic clinic of Sapienza University of Rome for metabolic evaluation. Patients were enrolled between 2000 and 2001 during routine clinical assessments (day hospital) with laboratory tests and upper abdomen ultrasound. Inclusion criteria were: diagnosis of T2D according to ADA criteria, male or female sex, age ≥ 18 years, no history of excessive alcohol consumption, as defined as daily alcohol intake exceeding 30 g for men and 20 g for women; negative results for hepatitis B surface antigen and hepatitis C virus antibody; absence of advanced liver disease, defined as hepatic decompensation or clinically significant portal hypertension; no history of other liver diseases (such as hemochromatosis, autoimmune hepatitis, or Wilson's disease); no current treatment with medications known to induce liver steatosis (e.g., corticosteroids, oestrogens, methotrexate, tetracycline, calcium channel blockers, or amiodarone) and availability of full clinical and laboratory data at baseline.

### 2.2 Clinical and Laboratory Evaluation

At baseline, all participants underwent a structured and standardised clinical evaluation. Anthropometric assessment included measurement of body weight (kg) and height (cm), with body mass index (BMI) calculated as weight divided by height squared (kg/m^2^). Waist circumference (cm) was measured at the midpoint between the lower margin of the last palpable rib and the top of the iliac crest using a flexible tape. Blood pressure was measured in a seated position after at least 5 min of rest using a validated automatic sphygmomanometer. The average of three consecutive readings was recorded for systolic (SBP, mmHg) and diastolic blood pressure (DBP, mmHg).

Detailed clinical history was collected by interview and medical record review, with specific attention to cardiovascular comorbidities, including documented ischemic heart disease, history of myocardial infarction, arterial hypertension (previous diagnosis or use of antihypertensive medication) and prior cerebrovascular events (stroke or transient ischemic attack).

Venous blood samples were drawn in the morning after a minimum of 12 h of overnight fasting. Biochemical analyses included: fasting plasma glucose (mg/dL), glycated haemoglobin (HbA1c, %), fasting serum insulin (μU/mL), total cholesterol (mg/dL), high‐density lipoprotein (HDL, mg/dL) cholesterol, low‐density lipoprotein (LDL, mg/dL) cholesterol and triglycerides (mg/dL), alanine aminotransferase (ALT, U/L) and aspartate aminotransferase (AST, U/L), all measured using standard enzymatic colorimetric methods. Renal function was assessed by measuring serum creatinine (mg/dL) and calculating the estimated glomerular filtration rate (eGFR, mL/min/1.73 m^2^) using the Chronic Kidney Disease Epidemiology Collaboration (CKD‐EPI) equation. Apolipoprotein B (ApoB, mg/dL) was quantified by immunoturbidimetric assay.

### 2.3 Hepatic Evaluations

Liver steatosis was assessed by conventional abdominal ultrasonography, performed in all participants by the same trained operator using a high‐resolution B‐mode scanner. The presence of steatosis was defined by characteristic sonographic criteria such as increased liver echogenicity, blurring of vascular margins and attenuation of the ultrasound, according to Saverymuttu et al. [21].

Metabolic dysfunction‐associated steatotic liver disease (MASLD) was diagnosed based on the presence of hepatic steatosis on ultrasonography, in the context of T2D as an established cardiometabolic risk factor, in accordance with the recent international consensus definition [22]. Liver fibrosis was estimated by calculating the FNI = e (−10.33 + 2.54 × ln(AST) + 3.86 × ln(HbA1c) − 1.66 × ln(HDL))/1 + e (−10.33 + 2.54 × ln(AST) + 3.86 × ln(HbA1c) − 1.66 × ln(HDL)) [17]. A cutoff value of FNI ≥ 0.33 was used to identify individuals at high risk of advanced fibrosis; values < 0.10 and 0.10 ≤ FNI < 0.33 indicated low and intermediate risk, respectively [14].

The association between estimated liver fibrosis and long‐term mortality was also explored by using other non‐invasive scores, such as the FIB‐4 [14, 15] and the AST to Platelet Ratio Index (APRI) [16] in age‐sex‐adjusted multivariable logistic regression models. FIB‐4 was calculated as follows: (Age* AST)/(Platelets *√(ALT)) and APRI according to the formula: (AST level/upper normal limit for AST)/platelet counts (10^9^/L) × 100. Upper normal limit for AST was set at 40 IU/L.

### 2.4 Follow‐Up and Outcome Definition

Participants were followed for 20 years. Mortality data were available for all study participants and were obtained through direct contact of family members, review of hospital records and consultation of civil registries. The follow‐up recall was conducted in 2021. The primary endpoint was all‐cause mortality.

### 2.5 Statistics

Statistical analyses were performed using SPSS version 28.0 (IBM Corp., Armonk, NY, USA). Continuous variables are expressed as mean ± standard deviation (SD) or median (25°–75° percentile) according to their distribution and categorical variables as frequencies and percentages. Between‐group differences were evaluated using the independent Student's *t*‐test or Mann–Whitney *U* test for continuous variables, depending on distribution and the chi‐square test for categorical variables. Logistic univariate and multivariate regression analysis was used to identify variables independently associated with 20‐year all‐cause mortality. Variables included in the multivariable model were selected based on clinical relevance and significance in univariate analyses. Odds ratios (ORs) and 95% confidence intervals (CIs) were reported. Statistical significance was set at a two‐sided *p*‐value < 0.05.

A *post hoc* power analysis was conducted to determine whether the study sample size was sufficient to detect differences in all‐cause mortality across fibrosis risk categories. On the basis of the observed mortality rates – 12.3% in the low‐risk group versus 47.7% in the moderate‐to‐high risk group – the study achieved a statistical power of 99.9% (α = 0.05), confirming that the sample was adequately powered to detect the observed effect size.

The study was conducted in accordance with the principles outlined in the Declaration of Helsinki and received ethical approval from the Ethics Committee of Policlinico Umberto I, Sapienza University of Rome, Rome, Italy. Participants provided written informed consent prior to study procedures.

## 3 Results

### 3.1 Baseline Evaluations

Our study cohort consisted of 174 individuals; 106 (60.9%) were men, mean ± SD age was 67.6 ± 11.4 years, median BMI was 28.6 (26–31.7) kg/m2 and diabetes duration was 5.5 (IQR 1–12) years. Glycemic control was acceptable (HbA1c 7.1% ± 1.7%). Overall, 78.2% were on antidiabetic agents and 16% on insulin therapy. Thirty‐two out of 174 (18.4%) had new‐onset diabetes (< 1 year). Baseline characteristics of the overall study population are detailed in Table 1.

**TABLE 1:** Baseline characteristics of the overall study population and according to 20‐year vital status.

Variable	Overall (*n* = 174)	Alive (*n* = 105)	Deceased (*n* = 69)	*p* ^a^
Age (years)	67.6 ± 11.4	63.43 ± 11.5	73.84 ± 7.9	< 0.001
Sex (male, %)	106 (60.9%)	60 (57.1%)	46 (66.7%)	0.271
Diabetes duration (years)	5.5 (1–12)	4 (1–11)	7 (2–16.2)	0.079
BMI (kg/m^2^)	28.6 (26–31.7)	29 (25.6–32.8)	28 (25.3–31.3)	0.356
Systolic BP (mmHg)	141.3 ± 18.2	137.87 ± 16.03	146.65 ± 20.15	0.004
Diastolic BP (mmHg)	81.1 ± 9.2	82.17 ± 7.92	79.53 ± 10.66	0.089
Hypertension (%)	124 (71.3%)	70 (66.7%)	54 (78.3%)	0.138
Smoking (%)	31 (17.8%)	18 (17.1%)	13 (18.8%)	0.933
Fasting glucose (mg/dL)	136.5 (114–161.2)	135 (112.8–156.8)	133 (115.5–157.8)	0.681
HbA1c (%)	7.1 ± 1.7	7.14 ± 1.72	7.16 ± 1.78	0.932
Insulin (μU/mL)	19.2 (14–27.7)	19 (14.1–29.4)	19.95 (13.45–25.7)	0.581
HOMA‐IR	6.8 (4.4–10.1)	6.6 (4.1–10)	5.6 (3.0–10.2)	0.844
Total cholesterol (mg/dL)	200.9 ± 57.8	201.76 ± 56.25	199.70 ± 60.49	0.818
HDL cholesterol (mg/dL)	45.2 ± 15.5	45.97 ± 15.68	43.93 ± 15.16	0.395
LDL cholesterol (mg/dL)	115.4 ± 37.6	117.68 ± 38.41	111.91 ± 36.22	0.347
Triglycerides (mg/dL)	143.5 (96–233)	151.5 (107–225.5)	156 (107–275)	0.406
ApoB (mg/dL)	116 (95.3–128)	111 (92–135)	112 (89–142)	0.656
Creatinine (mg/dL)	1.1 ± 0.4	1.05 ± 0.20	1.21 ± 0.61	0.014
eGFR (mL/min/1.73 m^2^)	76.3 ± 18.1	79.27 ± 16.99	71.67 ± 18.84	0.007
ALT (U/L)	26 (19–37)	27 (19–39)	24 (17–38)	0.103
AST (U/L)	23 (18–32.2)	23 (18–32)	25 (20–33)	0.789
Angina (%)	23 (13.3%)	9 (8.6%)	14 (20.3%)	0.041
Myocardial infarction (%)	32 (18.5%)	12 (11.4%)	20 (29.0%)	0.006
Stroke (%)	14 (8.0%)	5 (4.8%)	8 (11.6%)	0.147
ASCVD (%)	57 (33.0%)	22 (21.0%)	35 (50.7%)	< 0.001
Statins (%)	60 (34.5%)	33 (31.4%)	27 (39.1%)	0.377
Antiplatelets (%)	55 (31.4%)	26 (25%)	29 (42%)	0.022
Antihypertensive therapy (%)	124 (71.3%)	60 (57.1%)	52 (75.4%)	0.022
Oral antidiabetic therapy (%)	136 (78%)	80 (76.2%)	56 (81.2%)	0.41
Insulin therapy (%)	28 (16.1%)	15 (14.3%)	13 (18.8%)	0.556
FNI risk category				
Low risk (FNI < 0.10)	40 (23.0%)	32 (30.5%)	8 (11.6%)	0.008
Intermediate risk (FNI: 0.10‐ < 0.33)	58 (33.3%)	30 (28.6%)	28 (40.6%)	
High risk (FNI ≥ 0.33)	76 (43.7%)	43 (41.0%)	33 (47.8%)	

*Note:* Data are presented as mean ± standard deviation, median (interquartile range), or number (percentage), as appropriate.

Abbreviations: ALT, alanine aminotransferase; ApoB, apolipoprotein B; AST, aspartate aminotransferase; BMI, body mass index; Diastolic BP, diastolic blood pressure; eGFR, estimated glomerular filtration rate; ESR, erythrocyte sedimentation rate; FNI, Fibrotic NASH Index; HbA1c, glycated haemoglobin; HDL cholesterol, high‐density lipoprotein cholesterol; HOMA‐IR, homeostatic model assessment of insulin resistance; LDL cholesterol, low‐density lipoprotein cholesterol; Systolic BP, systolic blood pressure.

^a^ Comparisons between alive and deceased subjects were performed using Student's *t*‐test or Mann–Whitney *U* test for continuous variables and chi‐square test for categorical variables.

Ultrasound‐assessed fatty liver was present in 136/174 patients (78%), including 14 (8%) mild, 59 (34%) moderate and 63 (36%) severe cases. Overall, 75/174 patients (43%) were classified as having high fibrosis risk according to the FNI score. Within the study cohort, 116 were in primary prevention and 58 in secondary prevention. High fibrosis risk (FNI ≥ 0.33) was observed in 50/116 (43.1%) patients in primary prevention and 25/58 (43.1%) in secondary prevention, with no significant difference between groups (χ^2^ *p* = 0.78).

### 3.2 Follow‐Up Data

**FIGURE 1:**
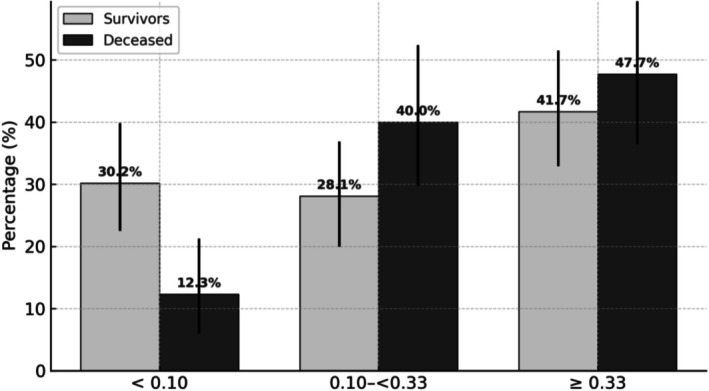
Distribution of 20‐year mortality according to Fibrotic NASH Index (FNI) risk categories. Bar plots show the proportion of deceased (dark grey) and surviving (light grey) individuals across the three FNI categories (< 0.10; 0.10 to < 0.33; ≥ 0.33). Differences between groups were assessed using the chi‐square test. *p = 0.025. FNI, Fibrotic NASH Index; SD, standard deviation. Categories were defined as follows: FNI < 0.10 = low risk, 0.10 ≤ FNI < 0.33 = intermediate risk, FNI ≥ 0.33 = high risk.

Over a 20‐year follow‐up, the all‐cause mortality rate in the cohort was 39.7% (69/174 patients). The distribution of FNI risk categories differed significantly between groups (*p* = 0.008; Table 1), with a higher proportion of moderate‐to‐high fibrosis risk among deceased individuals. Accordingly, the proportion of deaths increased across FNI categories, from 12.3% in the low‐risk group to 47.7% in the high‐risk group (*p* = 0.025; Figure 1).

### 3.3 Univariate and Multivariable Models for 20‐Year All‐Cause Mortality

At univariate logistic regression analysis, moderate‐to‐high fibrosis risk (FNI > 0.10; OR = 3.08, 95% CI: 1.31–7.28; *p* = 0.010), age (OR = 1.12, 95% CI: 1.07–1.17; *p* < 0.001) and eGFR (OR = 0.975, 95% CI: 0.957–0.994; *p* = 0.009) were associated with 20‐year mortality (Table 2).

**TABLE 2:** Logistic regression analyses for the association between fibrosis scores and 20‐year all‐cause mortality.

Variable	Univariate OR (95% CI)	*p*	Model 1 (FNI) OR (95% CI)	*p*	Model 2 (FIB‐4) OR (95% CI)	*p*	Model 3 (APRI) OR (95% CI)	*p*
FNI	3.08 (1.31–7.28)	0.01	11.75 (2.11–65.50)	< 0.001	—	—	—	—
FIB‐4	2.81 (1.54–5.11)	< 0.001	—	—	1.50 (0.52–4.37)	0.45	—	—
APRI	1.49 (0.29–7.53)	0.63	—	—	—	—	1.49 (0.29–7.53)	0.63
Age	1.12 (1.07–1.17)	< 0.001	1.27 (1.14–1.42)	< 0.001	1.21 (1.11–1.34)	< 0.001	1.22 (1.11–1.34)	< 0.001
Sex	1.50 (0.79–2.82)	0.21	1.79 (0.42–7.70)	0.44	3.50 (0.91–13.47)	0.07	3.44 (0.90–13.21)	0.07
BMI	0.97 (0.91–1.03)	0.38	1.07 (0.94–1.22)	0.30	1.06 (0.94–1.21)	0.35	1.07 (0.95–1.22)	0.26
ApoB	1.00 (0.99–1.01)	0.65	1.02 (1.00–1.04)	0.09	1.02 (1.00–1.05)	0.02	1.02 (1.00–1.05)	0.02
eGFR	0.97 (0.95–0.99)	0.01	1.04 (1.00–1.09)	0.07	1.02 (0.98–1.06)	0.39	1.02 (0.98–1.06)	0.35
Steatosis	1.04 (0.76–1.43)	0.12	1.12 (0.66–1.92)	0.67	1.35 (0.82–2.23)	0.24	1.33 (0.80–2.21)	0.28
ASCVD	3.88 (1.99–7.56)	< 0.001	2.94 (0.75–11.43)	0.12	2.13 (0.61–7.45)	0.24	2.03 (0.57–7.15)	0.27
Diabetes duration	1.03 (1.00–1.07)	0.07	0.96 (0.89–1.03)	0.24	0.97 (0.91–1.04)	0.40	0.97 (0.91–1.04)	0.39

*Note:* Odds ratios (ORs) and 95% confidence intervals (CIs) were estimated using univariate and multivariable logistic regression models. Model 1 includes FNI; Model 2 includes FIB‐4; Model 3 includes APRI. Multivariable models were adjusted for age, sex, BMI, ApoB, eGFR, steatosis, ASCVD, and diabetes duration. *p* values were derived from logistic regression analysis.

In multivariable models adjusted for age, sex, eGFR and potential confounders, thst is, BMI, ApoB, US‐detected fatty liver, diabetes duration, ASCVD, the FNI remained associated with 20‐year mortality (OR = 11.75, 95% CI: 2.11–65.49; *p* = 0.005), whereas FIB‐4 and APRI showed no independent association (Table 2).

Sixty‐six per cent of the study participants were ≥ 65 years at recruitment; in this subgroup, FNI persisted associated with 20‐year mortality in the same adjusted model as in the overall population (Table S1).

### 3.4 Liver Fibrosis, Mortality and Insulin Resistance

**FIGURE 2:**
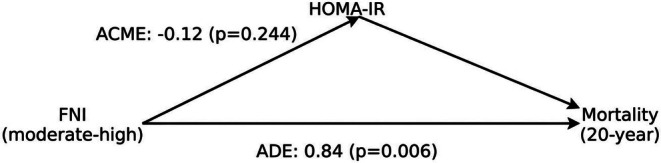
Mediation analysis of the association between fibrosis risk and 20‐year all‐cause mortality. Moderate‐to‐high fibrosis risk (FNI > 0.10) was modelled as the exposure, HOMA‐IR as the mediator, and 20‐year all‐cause mortality as the outcome. Mediation analysis was performed using a causal mediation framework based on regression models. The indirect effect (average causal mediation effect, ACME) was not statistically significant (estimate = −0.12; *p* = 0.244), whereas the direct effect (average direct effect, ADE) remained significant (estimate = 0.84; *p* = 0.006). The total effect was also significant (estimate = 0.72; *p* = 0.008).

To assess whether the association between fibrosis risk and 20‐year all‐cause mortality was mediated by insulin resistance, a mediation analysis was performed using HOMA‐IR as the mediator, moderate‐to‐high fibrosis risk as the exposure and mortality as the outcome. No significant indirect effect of FNI on mortality through insulin resistance was found, while the direct effect remained significant (Figure 2).

## 4 Discussion

This study investigated the association between liver fibrosis risk, as estimated by the FNI and mortality in a well‐characterised cohort of patients with T2D followed over two decades. Our results demonstrate that FNI‐estimated moderate‐to‐high fibrosis risk is independently associated with all‐cause mortality, irrespective of established cardiovascular risk factors or history, renal function and diabetic complications. Previous longitudinal studies have shown that commonly used non‐invasive fibrosis scores, such as FIB‐4 and NFS, retain a moderate ability to predict long‐term outcomes including overall mortality and liver‐related events, although their prognostic performance may be limited across different clinical settings and extrahepatic outcomes, particularly in metabolically complex populations [23]. In our investigation, FNI, but not FIB‐4 or APRI, retained a statistically significant association with the mortality outcome when adjusted for traditional risk factors.

While liver fibrosis is widely recognised as the strongest histological determinant of outcomes in MASLD [7, 24] and associated with poor metabolic control and increased prevalence of both microvascular and macrovascular diabetes' complications [6, 9, 10, 25, 26, 27], this study specifically highlights the prognostic relevance of FNI, an accurate, age‐independent non‐invasive marker, in a high‐risk T2D population. Despite being conducted in a cohort with a very high cardiovascular burden – where one‐third of participants were in secondary prevention – FNI emerged as the most significant determinant of long‐term mortality in adjusted models. Notably, baseline FNI values did not correlate cross‐sectionally with the presence of ASCVD, diabetic retinopathy, or renal disease, suggesting that FNI captures broader systemic risk beyond conventional diabetes‐related complications.

The absence of a cross‐sectional association between FNI value and micro‐ or macrovascular diabetes' complications at baseline, despite its strong predictive value for long‐term all‐cause mortality, may reflect the broader systemic implications of liver fibrosis. Hepatic fibrosis reflects chronic metabolic derangement, immune dysregulation and subclinical inflammation, all of which may progressively contribute to long‐term outcomes [28, 29]. Supporting this, several population‐based studies have shown that non‐invasive fibrosis scores – such as FIB‐4 and NFS – are associated with increased all‐cause mortality in T2D, even in individuals without cardiovascular disease at baseline [28, 29]. Leite et al. demonstrated that non‐invasive indexes of liver fibrosis independently predict all‐cause mortality in T2D, but neither the NFS nor the FIB‐4 were predictors of cardiovascular events or cardiovascular mortality, in line with our findings [29]. Furthermore, the association between liver fibrosis and adverse outcomes in diabetes appears to be stronger in the long term, suggesting that liver fibrosis may enhance metabolic disruption and exert detrimental clinical effects over time, more than just representing a marker of diabetes' complications or comorbidities. Taken together, these findings support the interpretation of liver fibrosis not only as a liver‐specific marker but as a global prognostic indicator in T2D, highlighting the importance of routine fibrosis screening in risk stratification. Despite this, liver fibrosis remains underdiagnosed in clinical practice, particularly among diabetic patients without overt liver disease [13].

Although numerous studies have shown that advanced fibrosis – whether assessed histologically or via non‐invasive indices – correlates with increased mortality, most have focused on populations with biopsy‐confirmed NAFLD and have relied on commonly used scores such as the NFS or the FIB‐4 [6, 7]. While these tools are well‐validated, their application in the diabetic population is limited by several constraints. In particular, FIB‐4 and NFS incorporate variables such as age, platelet count, or serum albumin, which may either confound risk estimation or reflect more advanced liver dysfunction, potentially reducing their sensitivity in detecting early‐stage disease [14, 15, 16]. Evidence on the long‐term prognostic implications of FNI is scarce. To our knowledge, no previous investigation has explored the relationship between FNI‐defined fibrosis risk and long‐term mortality in patients with T2D. Thus, the present study extends the evidence of an independent association between liver fibrosis and mortality risk in a diabetic cohort using the FNI, a marker specifically developed to reflect fibrotic burden in MASH and that has shown to outperform FIB‐4 in dysmetabolic cohorts at low risk of advanced liver damage [17, 18, 19].

Recent evidence indicates that the diagnostic accuracy of non‐invasive fibrosis tests is reduced in subjects with type 2 diabetes, due to both metabolic‐related alterations in biomarker levels and decreased specificity [30]. This highlights the need for tailored approaches in this population; within this framework, FNI may represent a practical and accessible tool for risk stratification in a large cohort of individuals at low risk of advanced liver disease. Moreover, FNI has demonstrated promising performance in dynamic clinical contexts, such as predicting fibrosis regression following bariatric surgery, suggesting that it may capture clinically relevant aspects of fibrosis burden beyond static cross‐sectional assessment [19]. However, it is important to acknowledge that most of the available evidence on FNI has focused on diagnostic accuracy or short‐ to mid‐term outcomes, while data on long‐term prognosis remain limited.

In a recent multicenter prospective study in patients with type 2 diabetes, the FNI showed good diagnostic performance for fibrotic MASH, although it was outperformed by imaging‐based scores such as FAST and MAST, supporting its role as a practical, non‐invasive, blood‐based tool while also underscoring the trade‐off between accessibility and diagnostic accuracy [31]. Starting from this point, our results extend current knowledge by showing that higher FNI values are independently associated with long‐term all‐cause mortality in a well‐characterised cohort of individuals with T2D, even after adjustment for multiple clinically relevant confounders. At the same time, in line with previous reports, we observed that other commonly used indices, including FIB‐4 and APRI, did not retain independent associations in fully adjusted models, supporting the notion that their prognostic utility may be attenuated in this specific population.

This study has some limitations that should be acknowledged. Although the number of events ensured adequate statistical power for multivariable analyses, the relatively small sample size, together with the wide confidence intervals, warrants cautious interpretation of the results and may limit their generalisability. Furthermore, follow‐up data were based on a single 20‐year recall, without precise information on mortality dates or causes of death. The absence of time‐to‐event data prevented the use of survival analyses, representing an additional important limitation of the study. Furthermore, the lack of cause‐specific data may have obscured distinct prognostic patterns and limited insights into the mechanisms linking fibrosis risk to long‐term outcomes. Nonetheless, the use of all‐cause mortality as the primary endpoint offers robustness and avoids classification bias. The absence of longitudinal treatment data also limits insight into evolving therapeutic effects; however, given that recruitment occurred in 2000 – prior to the availability of disease‐modifying antidiabetic agents – most patients were treated with older drugs not known to influence hepatic or cardiovascular outcomes. This context minimises the risk of confounding due to treatment changes. Moreover, the wide confidence interval associated with the FNI predictive value for long term mortality in our study population may reflect statistical imprecision, likely due to the limited number of deaths in the low‐ and intermediate‐risk groups. While the association with mortality remains significant, the exact magnitude of risk should be interpreted with caution. Larger studies are needed to confirm these findings and provide more precise risk estimates. In addition, our analysis was based on a single baseline FNI measurement, with no available data on changes in fibrosis status during the 20‐year follow‐up. Moreover, since liver biopsy was not clinically indicated in this cohort, histological data on steatosis, inflammation and fibrosis were not available. These limitations may have reduced the precision in characterising liver disease severity and progression, potentially affecting the strength of the observed associations. Finally, due to the lack of systematically collected data on the exact timing of death, a time‐to‐event analysis could not be performed. This limitation prevented the use of survival models and should be considered when interpreting the long‐term prognostic value of FNI and may also partly explain the lack of statistical significance observed for other established risk factors, such as BMI, ASCVD, eGFR and diabetes duration, whose prognostic impact may vary over time and be more accurately captured in time‐to‐event models. The absence of sociodemographic data, such as educational level, represents a limitation, as such variables may independently influence long‐term mortality and could not be accounted for in this analysis.

Nonetheless, this investigation has several strengths. It is based on a single‐centre cohort, followed over two decades by the same clinical team. All baseline data were collected using standardised protocols and analysed in a central laboratory, enhancing data quality and internal consistency. The population was well‐characterised, with a high prevalence of steatosis and fibrosis and an elevated long‐term mortality rate, providing an ideal setting for prognostic evaluations. Importantly, fibrosis risk, as assessed by an age‐independent marker, outperformed classical cardiovascular predictors, reinforcing the need to recognise liver fibrosis as a critical prognostic factor in T2D.

In conclusion, in T2D patients with high cardiovascular burden, FNI‐estimated hepatic fibrosis emerged as a major determinant of long‐term mortality. These findings support the early identification of liver fibrosis, using non‐invasive tools such as FNI, as a valuable clinical strategy to enhance risk stratification and guide personalised care in dysmetabolic populations.

## Author Contributions

I.B. and M.G.C. conceived the study; S.D., A.O., M.N.H., G.P., M.C. and M.A. collected the data and conducted the follow‐up. I.B. and F.A.C. performed data analysis. M.G.C. supervised the study. I.B. drafted the manuscript, and all authors (I.B., F.A.C., S.D., G.P., A.O., M.N.H., M.C., M.A., M.G.C.) critically revised the manuscript for important intellectual content and approved the final version.

## Funding

This work was supported by Ateneo Grants from Sapienza University (I.B., M.G.C.) and this research was funded by a PRIN grant from the Italian Ministry of University and Research (MUR).

## Ethics Statement

The study was conducted in accordance with the principles outlined in the Declaration of Helsinki and received ethical approval from the Ethics Committee of Policlinico Umberto I, Sapienza University of Rome. All participants provided written informed consent prior to enrolment.

## Conflicts of Interest

The authors declare no conflicts of interest.

## Supporting information


**Table S1:** Multivariable logistic regression model examining independent predictors of 20‐year all‐cause mortality in individuals ≥ 65 years old at the baseline.

## Data Availability

The data that support the findings of this study are available from the corresponding author upon reasonable and justified request.
