# Supplementary material for: Fibrotic NASH Index (FNI) Is Associated with Long‐Term Mortality in Individuals with Type 2 Diabetes and MASLD
Source: Liver Int. 2026 May 13;46:e70676. doi: 10.1111/liv.70676 (PMC13172655; doi:10.1111/liv.70676)
Supplement: Supplementary file 1 — Table S1: Multivariable logistic regression model examining independent predictors of 20‐year all‐cause mortality in individuals ≥ 65 years old at the baseline. [file LIV-46-0-s001.docx]

**Supplementary data**

**Supplementary Table 1S. Multivariable logistic regression model examining independent predictors of 20-year all-cause mortality in individuals ≥65 years old at the baseline.**

| **Variable** | **β** | **OR** | **95% CI (Lower)** | **95% CI (Upper)** | **p-value** |
| --- | --- | --- | --- | --- | --- |
| Moderate to high fibrosis risk (FNI > 10) | 2.701 | 14.901 | 1.760 | 126.145 | 0.013 |
| Age | 0.417 | 1.517 | 1.206 | 1.909 | <0.001 |
| Sex (male vs. female) | 0.014 | 1.015 | 0.179 | 5.740 | 0.987 |
| US fatty liver | 0.527 | 1.694 | 0.859 | 3.341 | 0.128 |
| BMI | 0.111 | 1.117 | 0.956 | 1.305 | 0.164 |
| ApoB | 0.020 | 1.020 | 0.994 | 1.047 | 0.130 |
| ASCVD | 0.051 | 1.052 | 0.995 | 1.112 | 0.075 |
| Diabetes’ duration | -0.037 | 0.964 | 0.891 | 1.041 | 0.349 |

*FNI is considered as a binary variable (moderate-to-high vs. low risk). US-assessed steatosis was modeled as an ordinal variable with semi-quantitative scale (0 = none, 1 = mild, 2 = moderate, 3 = severe). ASCVD was treated as binary (presence vs. absence). All other variables are included as continuous predictors.*
